# Authentic Allyship for Trans and Gender Diverse People: A Patterns of Knowing Perspective in Nursing

**DOI:** 10.1111/nup.70109

**Published:** 2026-08-02

**Authors:** John Gilmore

**Affiliations:** ^1^ School of Nursing, Midwifery and Health Systems University College Dublin Belfield Dublin Ireland

**Keywords:** authentic allyship, gender diverse care, nursing theory, patterns of knowing, transgender health

## Abstract

Trans and gender diverse people experience persistent inequities across healthcare systems, including discrimination in care, barriers to access, and poorer mental and physical health outcomes. While nurses are well placed to influence the quality and inclusivity of healthcare encounters, responses to these inequities have often focused primarily on improving clinical knowledge. Such an emphasis, while important, is insufficient to account for the relational, ethical, and structural conditions that shape care for trans and gender diverse communities. This paper draws on Carper's patterns of knowing, extended through Chinn and Kramer's emancipatory knowing, to reconsider authentic allyship in nursing. Using the patterns of empirical, aesthetic, personal, ethical, and emancipatory knowing, the paper argues that allyship is not reducible to technical competence, respectful intention, or symbolic inclusion. Rather, it requires the integration of evidence‐based understanding, compassionate and contextually attuned care, critical self‐reflection, moral responsibility, and structural awareness. Through this framework, the paper examines how nursing responses to trans and gender diverse health inequities can move beyond fragmented or individualised approaches towards a more reflective, socially responsive praxis. The paper further considers implications for nursing education, professional leadership, and organisational practice, arguing that authentic allyship must be embedded across curricula, clinical environments, and disciplinary commitments to social justice. Integrating patterns of knowing provides nursing with a multidimensional framework for practice that is not only clinically competent but also affirming, ethically grounded, and attentive to the structural conditions that shape health and healthcare.

## 1 Introduction

Trans and gender diverse individuals experience significant health inequities across healthcare systems internationally. These inequities manifest in multiple ways, including barriers to accessing healthcare services, discrimination within clinical environments, and poorer physical and mental health outcomes compared with cisgender populations (Cicero et al. 2019; White Hughto et al. 2015). Mental health disparities are particularly pronounced, with research consistently demonstrating higher rates of depression, anxiety, and suicidal ideation among trans populations, which are strongly associated with experiences of stigma and social exclusion (Cicero et al. 2019; Pinna et al. 2022; Hainey et al. 2025).

Healthcare environments play a critical role in shaping the experiences of trans and gender diverse individuals. Many patients report negative encounters with healthcare professionals, including misgendering, intrusive questioning unrelated to clinical care, and a lack of knowledge among providers regarding gender diversity and gender affirming healthcare (Alpert et al. 2023; Gilmore and Field 2026). Such experiences contribute to reduced trust in healthcare systems and avoidance of healthcare services (Allison et al. 2021).

Structural factors within healthcare systems also contribute to inequities. Administrative systems, documentation processes, and electronic health records frequently operate on binary assumptions about gender (Gilmore and Khan 2026; Streed et al. 2020). These structures can create barriers for individuals whose identities, bodies and expressions do not align with traditional categories of male or female. As a result, trans and gender diverse patients may encounter difficulties when attempting to access services or have their identities, bodies, or expressions accurately recognised within healthcare settings.

Nurses play a central role in shaping healthcare experiences. As the largest professional group within healthcare and often the primary point of contact for patients, nurses influence both the delivery and the culture of care. Nursing practice involves sustained engagement with patients across diverse settings, including hospitals, primary care services, and community environments. Through these interactions, nurses can contribute to healthcare environments that are either inclusive or exclusionary.

Educational responses to trans health disparities have increasingly focused on improving healthcare professionals' knowledge of gender diversity and gender affirming care. While improving knowledge is essential, research suggests that knowledge alone is insufficient to address inequities in healthcare experiences (Gilmore et al. 2026; Sherman et al. 2023; McCann and Brown 2020).

Allyship has emerged as an important concept in discussions of equity within healthcare. Authentic allyship refers to actions undertaken by individuals in positions of relative privilege to support marginalised communities and challenge inequitable systems. Within nursing practice, authentic allyship requires a commitment to listening to marginalised voices, reflecting on personal and professional assumptions, and advocating for structural change (Gilmore et al. 2024).

The epistemological framework of patterns of knowing in nursing, first introduced by Barbara Carper in the 1970s provides a useful lens for examining the forms of knowledge required for authentic allyship. Patterns of knowing recognise that nursing practice involves scientific knowledge, relational awareness, ethical reasoning, and reflective self‐understanding (Carper, 1978). Later developments within nursing theory introduced emancipatory knowing, which emphasises recognition of structural inequities and social justice (Chinn and Kramer 2007).

This paper draws on patterns of knowing to explore how nurses can cultivate authentic allyship and compassionate care for trans and gender diverse communities. By examining empirical, aesthetic, personal, ethical, and emancipatory knowing, the paper proposes a multidimensional framework for inclusive nursing practice.

### 1.1 Patterns of Knowing in Nursing

Nursing knowledge encompasses multiple ways of understanding health, illness, and the lived experience of those we care for. Patterns of knowing were first conceptualised by Barbara Carper (1978) as a framework to describe the epistemological foundations of nursing practice and to challenge an overly narrow emphasis on ‘scientific knowledge’ alone. Subsequent scholarship has expanded this framework, with Chinn and Kramer (2007) adding the concept of emancipatory knowing, emphasising that nursing knowledge must be understood as broad, value‐laden, and grounded in practice. These perspectives recognise that knowledge in nursing extends beyond empirical evidence and includes relational, ethical, reflective, and emancipatory forms of understanding (Porter 2010; Thorne 2020).

Rather than representing discrete or competing forms of knowledge, these patterns are interdependent and enacted simultaneously in practice. As highlighted by Chinn et al. 2018), nursing practice is most effective when these patterns are integrated, enabling praxis that responds not only to immediate patient needs but also to broader social contexts. Applying this framework to trans and gender‐diverse healthcare highlights the complexity of knowledge required to deliver care that is not only clinically competent but also authentic, inclusive, and socially responsive.

### 1.2 Empirical Knowing

As the scientific foundation of nursing practice, empirical knowing is indispensable in understanding the health needs, disparities, and interventions relevant to trans and gender diverse communities. Empirical knowing is often privileged within healthcare systems because it aligns closely with evidence‐based practice, clinical guidelines, measurable outcomes, and institutional expectations of competence. In the context of trans healthcare, however, the significance of empirical knowing is twofold. It is essential because nurses require a sound evidence base to deliver safe and effective care. Yet it is also limited because the existence of evidence alone does not ensure that care will be affirming, equitable, or compassionate. Empirical knowing must be treated as both foundational and incomplete.

A first empirical priority is the scale and patterning of trans health inequities. Evidence on the disease burden among trans populations globally demonstrates that trans and gender diverse individuals experience significant disparities across mental health, substance use, communicable diseases, and non‐communicable conditions (Gilmore et al. 2026; Scheim et al. 2024). However, the global understanding of this burden remains fragmented. There are substantial methodological and geographical gaps in the evidence, with much of the research concentrated in particular high‐income regions and underdeveloped engagement with community‐informed or participatory methodologies (Gilmore 2024; Scheim et al. 2024). This uneven distribution of knowledge constrains the ability to fully understand the diversity of trans health experiences across different cultural and healthcare contexts.

These limitations are further compounded by the historical and ongoing exclusion of gender identity within health data systems. In many healthcare settings, gender identity is either not routinely collected or is recorded in ways that are inconsistent, binary, or clinically unusable (Gilmore and Khan 2026; Streed et al. 2020). This lack of integration of gender identity data within electronic health records and population health datasets restricts the visibility of trans populations within health surveillance and epidemiological research. As a result, trans health inequities may be underestimated, misclassified, or rendered invisible within national and global health statistics. This absence is not neutral; it reflects broader structural marginalisation and reduces the capacity of healthcare systems to respond effectively to identified needs.

Importantly, the disparities that are evident in the available evidence must not be interpreted as intrinsic to trans identity. Rather, they should be understood within the broader context of oppression, intersectional stigma, barriers to care, and systemic discrimination (Hendricks and Testa 2012). This aligns with minority stress frameworks, which conceptualise health inequities as arising from chronic exposure to social stressors rather than from identity itself (Meyer 2003; Hendricks and Testa 2012). This distinction is critical. It shifts the focus of empirical knowing away from individual pathology and towards the social and structural conditions that shape health outcomes. Empirical knowing in trans healthcare must extend beyond a narrow biomedical frame to include evidence related to social determinants of health, stigma, and structural exclusion.

A second empirical priority concerns the evidence base for gender‐affirming care itself, the medical and surgical interventions which support an individual to alter their physical gender expression. This evidence base remains relatively emergent, shaped by the historical marginalisation of trans health within research, the exclusion of gender identity within routine data collection, and increasingly restrictive legal and policy environments that influence both access to care and the feasibility of conducting research. Cooney et al. (2025), in their systematic review of twenty‐eight studies, including four randomised controlled trials and 24 longitudinal studies on health and quality of life outcomes, values and preferences, and costs associated with gender‐affirming care, found that such care may improve mental health, quality of life, stigma‐related outcomes, and healthcare utilisation, with no significant harms identified in the studies included. These findings are particularly important in contemporary healthcare contexts where gender‐affirming care is sometimes misrepresented as ideologically driven rather than evidence‐informed.

An incomplete or developing evidence base should not be conflated, however, with evidence of ineffectiveness or harm. In trans healthcare, what is often positioned as the gold standard of evidence, particularly randomised controlled trials, is not always feasible, ethical, or methodologically appropriate for evaluating medical and surgical gender‐affirming interventions. As Noone et al. (2025) argue, rigid applications of evidence hierarchies can be problematic in this highly politicised area, particularly where observational and longitudinal data, which may be more appropriate to the research context, are discounted. They further highlight methodological inconsistencies and potential bias in how evidence relating to gender‐affirming care is appraised and mobilised, raising concerns about double standards in the evaluation of supportive versus restrictive evidence. Emerging evidence also demonstrates that uncertainty regarding access to gender‐affirming care has important health implications, with many transgender adults reporting intentions to seek unregulated alternatives or experiencing suicidal ideation should access be restricted (Graziano 2025).

A further important empirical distinction is between gender‐affirming care and inclusive healthcare more broadly. Cooney et al. (2025) clearly differentiate gender‐affirming care, which may include hormones, surgery, and psychosocial interventions, from inclusive care practices, which include respectful terminology, inclusive facilities, and structural changes that support access. This distinction is important, while in many contexts a nurse may not prescribe hormones or perform surgery, but every nurse participates in inclusive or exclusionary care practices. Empirical knowing must extend beyond the specific treatments often associated with gender‐affirming care and include knowledge of the environmental, linguistic, and organisational factors that shape access and patient experience.

For example, a nurse caring for a trans man attending primary care for cervical screening requires empirical knowledge that the presence of a cervix, rather than gender identity, determines screening need. Without this knowledge, opportunities for preventive care may be missed. Empirical knowing enables nurses to move beyond assumptions and deliver care based on clinical evidence rather than gender stereotypes.

For nurses, empirical knowing requires more than familiarity with available research. It involves critically engaging with how evidence is produced, recognising the limitations of dominant evidence hierarchies, and understanding how structural, methodological, and political factors shape what counts as knowledge. Gender‐affirming care is not a symbolic gesture detached from health outcomes. It is an area of care increasingly supported by evidence as beneficial for those who seek it, and its appraisal must take account of the methodological, ethical, and contextual realities of research in this field.

At the same time, the nursing‐specific evidence base remains underdeveloped. Existing research highlights a continued lack of primary studies focused on nursing care delivery for transgender people, as well as gaps in nursing education related to gender‐affirming care (Kimmel et al. 2024; Medina‐Martínez et al. 2021). Despite increasing visibility of trans health issues, this incomplete evidence base constrains the development of nursing knowledge and practice in this area. This matters because nursing occupies a distinctive place within healthcare. Nurses spend more time with patients than many other professionals, coordinate care, interpret systems, and shape how care is experienced at the bedside. If the nursing evidence base is weak, then nurses may be left to practise in areas of high relational and ethical complexity without adequate disciplinary guidance.

### 1.3 Aesthetic Knowing

Carper (1978, p. 16) characterised aesthetic knowing as ‘abstracted particulars,’ referring to the nurse's ability to grasp the unique features of a situation through an immediate understanding of what is meaningful in the moment. This form of knowing is not derived from generalisable rules or formal reasoning, but from a direct engagement with the situation itself. It is the ‘art of nursing’ (Chinn et al. 2018, p.24). Meaning is often shared between those in the encounter without explicit verbal exchange and may not be consciously articulated, instead operating in the background as an embodied and relational form of understanding. The nurse's capacity to grasp the meaning of a clinical situation, recognise what matters in the moment, and respond in a way that is humane, contextually attuned, and therapeutically significant.

In the context of trans and gender diverse healthcare, aesthetic knowing is indispensable because so much of the harm reported by trans patients occurs not only through lack of treatment or structural exclusion, but through the felt quality of encounters (Gilmore and Field 2026). Patients may be technically treated yet still feel erased, scrutinised, disbelieved, or exposed. Aesthetic knowing is what enables nurses to perceive this dimension of care and to act in ways that make healthcare feel safe, respectful, and affirming.

Small interventions such as listing one's own pronouns, using gender‐neutral language, validating and affirming the patient, and utilising appropriate mental and physical health screening can significantly influence patient experience, health outcomes, and quality of life (Bhatt et al. 2022). Although these recommendations might at first appear procedural, they are deeply aesthetic in Carper's sense. They require nurses to notice how the patient is situated emotionally and socially in the encounter and to respond in a manner that communicates recognition rather than threat. Aesthetic knowing is therefore not superficial kindness. It is a disciplined relational competence.

Trans and gender diverse patients often enter healthcare spaces carrying prior experiences of stigma or anticipating discrimination. Trans patients are subject to microaggressions, misgendering, and harassment from providers, staff, and other patients (Bhatt et al. 2022; Cicero et al. 2019; Gilmore and Field 2026). These realities shape how an encounter is interpreted from the moment it begins. Aesthetic knowing means recognising that a routine question, a pause, a misused pronoun, or an unnecessary glance can carry disproportionate weight in this context. The nurse who is aesthetically attuned does not treat these as minor slips in etiquette. They understand them as cues that affect whether the patient feels safe enough to remain present, disclose concerns, or trust the care being offered.

García‐Acosta et al. 2024 reinforce this through their systematic review of safe and inclusive healthcare environments for transgender people. Their findings are organised around the training of health professionals, the creation of safe spaces, the nurse as facilitator, and best care practice. Across these domains, they emphasise warmth, respect, inclusivity, use of neutral language, and recognition of a person's preferred name and pronouns. These elements can be read as institutional recommendations, but they are equally descriptions of the aesthetic quality of good care. A healthcare environment communicates something even before a word is spoken. Aesthetic knowing allows nurses to see that communication and influence it. It encompasses not only how the nurse speaks and behaves, but also how they mediate the patient's relationship with the wider setting.

One important feature of aesthetic knowing is its attentiveness to embodiment and vulnerability. For example, a transmasculine person presenting for routine care may also be managing significant chest dysphoria. Graziano 2023 meta‐ethnography illustrates how seemingly ordinary clinical interactions can become psychologically distressing when clinicians fail to understand the meaning that particular body characteristics hold for patients or rely on stereotypical assumptions about gender.

Bhatt et al. (2022) highlight the need for sensitive approaches to screening, physical examination, and the discussion of anatomy or sexual health. In trans healthcare, bodily care is often emotionally charged. Parts of the body may be associated with dysphoria, shame, social anxiety, or previous traumatic encounters. A nurse acting only from empirical knowing may understand what screening is required. A nurse drawing on aesthetic knowing understands how to approach that screening. They explain why it is necessary, seek consent with care, attend to the patient's affect, and pace the encounter in ways that preserve dignity. This is the art of nursing in a highly concrete sense.

Aesthetic knowing is also evident in how nurses respond to systems that are not built for the patient. Electronic records and administrative systems may not accommodate correct names and pronouns, yet nurses still make choices within those barriers (Bhatt et al. 2022; Gilmore and Khan 2026). One professional may reproduce the system's misrecognition without reflection, while another may manually correct language, acknowledge the patient respectfully, and explain the constraints of the record while affirming the person before them. The technical problem may be the same, but the aesthetic difference is profound. One approach compounds harm. The other softens it and communicates alliance.

Consider a trans woman admitted to a medical ward who notices staff repeatedly referring to her by the incorrect name displayed on an electronic record. While the technical error may be outside the nurse's immediate control, the nurse can acknowledge the mistake, use the patient's affirmed name and pronouns, and explain the limitations of the system. The clinical intervention may be unchanged, but the experience of care is profoundly different. Aesthetic knowing allows the nurse to recognise and respond to the relational significance of these moments.

Caring for individuals undergoing gender‐affirming surgery highlights how the quality of nursing encounters is shaped by factors such as limited knowledge, communication challenges, environmental constraints, and underlying biases. Even in the absence of explicitly negative attitudes, care may still be influenced by uncertainty and unexamined assumptions, shaping how patients experience the interaction (Peynirci and Mecdi Kaydırak 2026). Good intentions alone do not ensure affirming care; aesthetic knowing requires practice, awareness, and the ability to perceive subtle meanings within clinical encounters. A nurse may consider themselves respectful, yet still contribute to an atmosphere of discomfort or conditional acceptance if these relational dimensions are not recognised and addressed.

Aesthetic knowing is closely linked to compassion, but compassion here should not be romanticised. It is not pity, nor is it an emotional excess that overrides professional boundaries; it is a mode of disciplined responsiveness to suffering, vulnerability, and personhood. For trans and gender diverse patients, compassion often takes the form of recognition; not having to fight to be named correctly, not having one's body treated as a curiosity, not being asked irrelevant or voyeuristic questions, and not being left to manage staff discomfort. The nurse who can provide this kind of encounter is doing more than being nice. They are enacting a form of skilled, person‐centred practice.

For authentic allyship, aesthetic knowing is critical because it is the pattern through which care becomes experientially trustworthy. Allyship cannot be reduced to ideological commitment or public declarations of inclusion. It must be perceptible to the patient in the encounter itself. In this sense, aesthetic knowing is one of the strongest bridges between Carper's framework and compassionate trans‐inclusive practice. It translates evidence into interaction and principle into atmosphere. Without it, trans care may remain technically informed but emotionally unsafe. With it, the nurse can create moments in which dignity is actively restored rather than merely presumed.

### 1.4 Personal Knowing

Personal knowing is perhaps the most complex and easily distorted of Carper's patterns. Chinn et al. 2018) describe personal knowing as the process of developing self‐awareness and authenticity within the therapeutic relationship, emphasising the importance of knowing oneself in relation to others and using that understanding to guide practice. This builds on Carper's (1978, p.18) original articulation of personal knowing as being concerned with ‘the knowing, encountering, and actualising of the concrete individual self, ’ a process that involves standing in relation to another human being and encountering that person as a person. It is expressed through the therapeutic use of self, where the nurse's values, experiences, and presence shape how care is enacted. However, unless carefully defined, personal knowing risks becoming an uncritical endorsement of personal beliefs, intuition, or comfort.

In the context of authentic allyship with trans and gender diverse communities, this distinction is essential. Personal knowing is not permission for nurses to foreground personal uncertainty, ideology, or moral discomfort as though these were professionally neutral. Rather, it is a disciplined process through which nurses come to understand how self enters practice, how implicit and explicit biases operate, and how their position of power may facilitate or obstruct access to safe, affirming care.

Thorne (2020) argues that personal knowing was originally intended as the discovery of self‐and‐other through reflection, synthesis of perceptions, and relational engagement, but cautions against its uncritical use as justification for personal beliefs that conflict with professional responsibilities. This critique has immediate relevance to trans healthcare, where issues of gender diversity are often socially and politically contested. Nurses may interpret discomfort, uncertainty, or disagreement as matters of personal belief, yet such positioning risks legitimising harmful or exclusionary practices. Importantly, implicit bias operates in ways that are often outside conscious awareness, shaping perceptions, communication, and decision‐making even when explicit intentions are affirming. Personal knowing requires more than reflection on stated beliefs; it requires active engagement with the less visible assumptions that influence practice and a willingness to interrogate how these may manifest in subtle but impactful ways within care encounters.

Nurses may hold both implicit and explicit biases towards transgender people, including a tendency to conflate sex and gender identity and to interpret trans experiences through binary or essentialist frameworks (Derbyshire and Keay 2023). These biases are not always consciously recognised and may persist even where explicit attitudes appear neutral or affirming, indicating that the issue extends beyond knowledge deficits to deeper attitudinal patterns that shape perception, communication, and decision‐making in practice. Without critical reflection, such biases may continue to operate beneath the surface, influencing interactions and interpretations in ways that are difficult for practitioners to detect but are readily experienced by those receiving care (Derbyshire and Keay 2023). Personal knowing becomes the mechanism through which implicit bias is brought into awareness and critically examined. Without this, biases may continue to operate unchallenged, influencing care in ways that are subtle but consequential, including misrecognition, avoidance, or differential engagement.

Moving reflection beyond simple introspection to accountability requires nurses not only to recognise bias but to consider how it shapes care and to take responsibility for repair where harm occurs (Lippe et al. 2023). This framing is particularly important in relation to implicit bias, which may not be immediately visible to the practitioner but can nonetheless be experienced by patients through tone, hesitation, body language, or patterns of interaction. Personal knowing involves an ongoing commitment to uncovering and addressing these dimensions of practice, rather than assuming that good intentions are sufficient.

In practice, personal knowing begins with examining assumptions about bodies, identity, language, and legitimacy. Nurses may assume that gender can be inferred from appearance, anatomy, or voice, or that uncertainty should be managed through avoidance rather than engagement. For example, during an admission assessment, a nurse may realise they are uncertain about a patient's gender identity and preferred pronouns. Rather than allowing this uncertainty or implicit assumptions to shape the interaction, personal knowing supports a reflective pause in which the nurse acknowledges the limits of their own knowledge and respectfully asks the patient how they wish to be addressed. The therapeutic use of self is evident not in having all the answers, but in responding with authenticity, humility, and openness to the person's own account of themselves. Implicit biases may shape expectations about patients' needs, behaviours, or credibility, even in the absence of conscious prejudice. These responses are not uncommon, but personal knowing requires that they are not left unexamined. Without this work, harm may be reproduced even when care is intended to be respectful. Personal knowing is therefore inseparable from an awareness of power. Nurses occupy positions of institutional authority, and for trans patients who may anticipate stigma or dismissal, this power shapes the encounter. Patients may assess whether the nurse is safe, whether they will be respected, or whether they will need to protect themselves. Recognising this, personal knowing requires an understanding that the nurse does not enter the encounter as a neutral individual, but as a professional whose actions carry significant relational and ethical consequences.

Personal knowing does not require complete mastery of trans healthcare, but it does require honesty about the insufficiency of knowledge without shifting the burden of education onto the patient. It also involves recognising emotional responses, including discomfort, uncertainty, or moral tension, and holding these reflectively rather than allowing them to shape care. Personal knowing also requires recognising when patients may be reluctant to disclose concerns because of previous experiences of stigma or anticipated discrimination, creating conditions in which they feel safe to discuss their goals for gender‐affirming care without fear of judgement (Graziano 2025). Importantly, implicit bias may be most influential in moments of uncertainty or discomfort, where automatic assumptions can guide behaviour. Professional maturity lies in the ability to pause, reflect, and respond deliberately, rather than defaulting to these unexamined patterns.

Personal knowing becomes a key distinction between authentic and performative allyship. Performative allyship may involve correct language or visible support without deeper self‐examination. In contrast, authentic allyship requires sustained critical reflection, a willingness to confront implicit and explicit biases, and accountability for how one's presence shapes care. Personal knowing is not a validation of personal perspective, but a discipline of self‐awareness in service of equitable practice.

### 1.5 Ethical Knowing

Chinn et al. 2018) describe ethical knowing as concerned with matters of obligation, responsibility, and moral judgement, expressed in practice through decisions about what is right, just, and responsible in specific situations. Ethical knowing is not an abstract moral philosophy; it is enacted in everyday practice through decisions that protect dignity, respond to vulnerability, negotiate confidentiality, challenge harm, and advocate for equitable care. In the context of trans and gender diverse healthcare, this pattern is particularly significant, as care is often delivered within systems that do not consistently support recognition, safety, or inclusion. Nurses have a role not only as clinical actors but also as moral agents who must decide how to respond when structures, practices, or colleagues undermine the personhood of the patient.

The International Council of Nurses Code of Ethics emphasises that nursing care must be delivered with respect for human rights, including cultural rights, the right to life and choice, and must be provided without discrimination of any kind (International Council of Nurses 2021). Core ethical principles such as beneficence, non‐maleficence, autonomy, and justice take on specific relevance when caring for trans and gender diverse patients. Providing trans‐inclusive care is not an optional extension of practice but is required to meet these ethical obligations.

As illustrated in nursing‐focused analyses of care for trans patients, failure to recognise or respond appropriately to identity and need risks causing harm, withholding benefit, and perpetuating inequity (Gilmore et al. 2025). Respect for autonomy includes recognising a patient's self‐identified gender, name, and pronouns, while misgendering or refusal of recognition constitutes a denial of the patient's authority over their own identity and personhood (Rodríguez et al. 2023). Ethical knowing also requires careful attention to confidentiality; decisions about what information is clinically necessary, who should have access to it, and how to protect privacy are particularly significant in trans care, where disclosure may expose patients to discrimination or unnecessary scrutiny (Gilmore et al. 2025). Nurses must therefore consider not only what should be known, but by whom, for what purpose, and with what potential consequences.

Ethical knowing is also inherently relational and responsive; it involves recognising when harm has occurred and taking responsibility for repair. As Lippe et al. (2021) argue, ethical care includes obligations to acknowledge mistakes, offer an apology, and rebuild trust when care has been experienced as harmful. In this sense, ethical knowing is not reduced to the avoidance of wrongdoing but includes the capacity to respond to harm in ways that support dignity and restore relational integrity. It also extends beyond individual interactions to the broader systems in which care is delivered. Trans patients frequently encounter inequitable environments, including documentation processes that fail to recognise identity, discriminatory language, and barriers to accessing gender‐affirming care (Cooney et al. 2025); nurses must recognise that restrictions on such care may contribute to avoidable psychological distress and poorer health outcomes (Graziano 2025). Ethical knowing requires nurses to move beyond passive awareness and actively challenge practices that perpetuate injustice (Gilmore et al. 2024).

Dilemmas in trans healthcare are rarely resolved through principle alone; they require judgement, courage, and sensitivity to context. Tensions may arise between confidentiality and clinical decision‐making, between respect for identity and inflexible systems, or between patient advocacy and organisational norms. Ethical knowing in these situations is not a checklist but a form of disciplined moral reasoning grounded in professional responsibility and patient‐centred care. It calls for the integration of ethical principles with situational awareness and relational understanding.

Ethical knowing gives allyship its normative force. Allyship that remains symbolic may endorse inclusion in principle while failing to act in moments of moral tension. Ethical knowing transforms this endorsement into an obligation, making clear that respect, dignity, and equity are integral to nursing practice rather than optional enhancements. It also calls forth moral courage, requiring nurses to intervene, challenge harmful behaviour, and advocate for change, even when doing so is uncomfortable. At a broader level, ethical knowing highlights that responsibility does not rest solely with individual practitioners. Ethically sound care requires adequate training, organisational support, and systems that enable rather than constrain inclusive practice (Gilmore et al. 2025; Rodríguez et al. 2023; Lippe et al. 2021).

Justice is perhaps the principle that most clearly connects ethical knowing to allyship. Trans patients frequently encounter inequitable systems, from documentation processes that do not recognise identity to environments where discriminatory language is used or where access to gender‐affirming care is obstructed. Injustice in this context is not an external or peripheral issue but a matter directly relevant to nursing practice. When colleagues mock, dismiss, or fail to recognise a trans patient, nurses are not passive bystanders; ethical knowing obliges them to recognise and challenge such practices, positioning advocacy and intervention as integral to professional responsibility (Gilmore et al. 2024).

### 1.6 Emancipatory Knowing

Emancipatory knowing, initially articulated by Chinn and Kramer (2007), is the pattern through which nurses recognise how social, political, institutional, and historical structures shape health and healthcare. It moves beyond the immediacy of the clinical encounter and beyond individual intentions, directing attention to the systems that produce inequity and to nursing's role in challenging them. Emancipatory knowing involves uncovering taken‐for‐granted assumptions, questioning who benefits from existing arrangements, and engaging in praxis, where critical reflection is linked to action (Chinn et al. 2018). For trans and gender diverse healthcare, this pattern is indispensable because inequities are not simply the result of isolated prejudice or individual deficit. They are reproduced through documentation systems, educational omissions, policy barriers, legal and insurance restrictions, pathologising histories, and healthcare environments organised around cisnormative assumptions. Authentic allyship, therefore, requires more than respectful interpersonal care. It requires structural awareness and a willingness to participate in change.

Evidence from trans health research demonstrates that health inequities and care experiences are fundamentally shaped by wider structural conditions. Disparities in trans health are closely linked to systemic oppression, intersectional stigma, discrimination, and barriers to care, rather than arising from individual factors alone, as articulated within minority stress frameworks (Hendricks and Testa 2012; Scheim et al. 2024; Gilmore et al. 2026). These structural forces shape not only health outcomes but also access to care, interactions within healthcare settings, and broader experiences of inclusion and exclusion. At the same time, inequities are reflected in the uneven development of the evidence base and in gaps within nursing knowledge and education, where trans health remains under‐integrated (Medina‐Martínez et al. 2021). From an emancipatory perspective, injustice is therefore evident not only in patterns of morbidity and access but also in whose health is prioritised, how knowledge is produced, and how care systems are organised.

These structural dynamics are reproduced within healthcare systems and everyday practice. Administrative processes that fail to recognise identity, non‐inclusive environments, and gaps in professional knowledge contribute to ongoing marginalisation (García‐Acosta et al. 2024; Gilmore and Khan 2026), while access to gender‐affirming care remains uneven despite growing evidence of its benefits for mental health and quality of life (Cooney et al. 2025).

Emancipatory knowing also has implications for action within healthcare systems, particularly in relation to advocacy. Emancipatory knowing is realised through praxis, where critical awareness of injustice is linked with efforts to transform the conditions that sustain it (Chinn et al. 2018). In this sense, nurses are not only providers of care but advocates who play a key role in shaping inclusive environments, policies, and practices, including the development of gender‐neutral facilities, the use of inclusive language, and engagement in organisational change (Bhatt et al. 2022; Gilmore et al. 2024). This work is central to addressing the structural conditions that produce inequity, moving beyond critique to the ongoing transformation of healthcare environments so that trans patients are not repeatedly required to negotiate recognition and legitimacy.

Intersectionality is also central to this pattern of knowing; experiences of inequity are not uniform for trans and gender diverse people, but shaped by intersecting forms of marginalisation, including race, class, disability, and migration status (Agénor et al. 2022; Wesp et al. 2019). These intersecting positions influence both how individuals experience health and how they encounter healthcare systems, often shaping access, recognition, and outcomes in uneven ways (Agénor et al. 2022).

Emancipatory knowing, therefore, requires nurses to move beyond generalised assumptions and attend to the ways in which different forms of disadvantage may converge in practice. This involves recognising that patients' experiences are shaped by multiple, overlapping influences rather than a single dimension of identity, and that healthcare responses may need to be sensitive to this complexity. In this way, intersectionality adds depth to emancipatory knowing by drawing attention to variation within experiences of inequity and the importance of context in shaping care. In practice, emancipatory knowing shifts the questions that guide nursing. Rather than asking only how to provide respectful care within existing systems, it asks what aspects of those systems produce exclusion, whose interests are served, and what can be changed. This reorientation moves allyship beyond individual virtue and towards institutional accountability and leadership, recognising that compassionate care cannot be fully realised without addressing the structures that undermine it.

Emancipatory knowing ensures that Carper's framework is not confined to the interpersonal level. It makes visible the connection between knowledge, power, and justice, and highlights the role of nursing in challenging inequity at both individual and structural levels. Without this pattern, nursing risks becoming skilled at offering compassionate care within inequitable systems while leaving those systems intact. With it, nursing can align its commitments to dignity, inclusion, and person‐centred care with the broader work required to make those commitments materially real.

### 1.7 Integrating Patterns of Knowing

Authentic allyship in nursing emerges through the integration of multiple patterns of knowing within practice, where empirical, aesthetic, personal, ethical, and emancipatory ways of knowing are enacted together in response to complex clinical and social realities. This integration reflects the understanding that nursing knowledge is not singular or hierarchical, but relational, contextual, and realised through practice (Chinn et al. 2018). Each pattern contributes a distinct dimension of understanding, yet it is their interdependence that enables care to be clinically competent, relationally attuned, ethically grounded, and socially responsive.

Consider a trans woman presenting to an emergency department with abdominal pain. Empirical knowing guides assessment and ensures clinical decisions are based on anatomy and presenting symptoms rather than assumptions about gender. Aesthetic knowing helps the nurse recognise the patient's anxiety after previous experiences of discrimination and respond in ways that foster trust. Personal knowing requires reflection on any assumptions or biases that may influence the encounter. Ethical knowing guides respect for the patient's identity, confidentiality, and autonomy, while emancipatory knowing draws attention to organisational barriers such as documentation systems that fail to recognise affirmed names and genders. When integrated, these patterns demonstrate how authentic allyship emerges not from any single form of knowledge, but from their integration in practice.

Empirical knowing provides the scientific foundation for understanding health disparities and the benefits of gender‐affirming care, while aesthetic knowing shapes how care is experienced through compassionate and contextually sensitive engagement. Personal knowing supports critical self‐awareness and reflexivity, enabling nurses to recognise and address implicit bias, and ethical knowing guides action through commitments to dignity, autonomy, and justice. Emancipatory knowing extends this further by situating care within broader structural conditions and highlighting the role of nursing in addressing inequity (Chinn and Kramer 2007; Chinn et al. 2018). These patterns do not operate in isolation; rather, they are mutually reinforcing. Without empirical grounding, care risks being uninformed; without aesthetic and personal knowing, it may be experienced as unsafe; without ethical knowing, it lacks moral direction; and without emancipatory knowing, it risks reproducing the very inequities it seeks to address. Their integration represents a form of praxis in which knowledge, reflection, and action are inseparable.

This integrated framework has important implications for nursing education. Preparing nurses for inclusive practice requires more than the addition of discrete content on trans health; it requires a reorientation of curricula to ensure that trans health is both embedded and explicitly addressed. Approaches such as usualising and specificising provide a useful framework, integrating trans health across core teaching while also addressing specific health needs and inequities, ensuring that it is neither marginalised nor treated as optional or specialist knowledge (Gilmore et al. 2026). Educational strategies that incorporate simulation, case‐based learning, reflective practice, and engagement with lived experience can support the development of relational, ethical, and critical dimensions of care (Koch et al. 2021; McCann and Brown 2020). In this way, the integration of patterns of knowing becomes not only a conceptual framework but a pedagogical imperative.

Professional nursing organisations have a key role in enabling this integration and in signalling that trans‐inclusive, socially just practice is a professional expectation rather than an individual preference. Organisations such as the American Nurses Association and the International Council of Nurses have explicitly positioned social justice as a core professional responsibility, reinforcing that nurses are expected to address inequities and advocate for marginalised populations (American Nurses Association 2017; International Council of Nurses 2021). This kind of professional leadership matters because it shapes standards, priorities, policy positions, and the wider culture of the profession. It also provides a collective mandate for nurses to engage in advocacy, challenge discrimination, and support system‐level change, rather than locating allyship solely at the level of personal values or isolated practice behaviours (Bhatt et al. 2022; Gilmore et al. 2024). In this sense, professional bodies do more than issue statements. They help define what counts as legitimate nursing knowledge, ethical practice, and professional responsibility.

## 2 Conclusion

Trans and gender diverse health inequities are not solely the result of individual interactions or isolated gaps in knowledge, but are shaped by broader social, structural, and institutional conditions that influence how care is accessed, delivered, and experienced. Within this context, nursing has a critical role to play, not only in providing care but in shaping healthcare environments, challenging inequity, and advocating for inclusive practice. This paper has argued that authentic allyship requires a form of nursing practice that extends beyond technical competence or declarative support for inclusion, and instead is grounded in a multidimensional understanding of knowledge.

Carper's patterns of knowing offer a valuable lens through which to conceptualise this work. When integrated, these patterns move nursing practice beyond fragmented or partial responses, enabling a form of praxis that is reflective, relational, and socially engaged (Chinn et al. 2018).

Ultimately, authentic allyship in nursing is not a fixed identity or a set of isolated actions, but an ongoing, reflexive, and practice‐based commitment. It requires nurses to engage with multiple ways of knowing, to critically examine their own assumptions, and to recognise and respond to the structural conditions that shape health and healthcare. For trans and gender diverse care, this means moving beyond symbolic inclusion towards practice that is consistently affirming, ethically grounded, and structurally aware. Through the integration of patterns of knowing, nursing is uniquely positioned to contribute to healthcare that not only treats illness but also promotes dignity, equity, and justice for all.

## Funding

The author has nothing to report.

## Ethics Statement

Ethical approval was not required for this study as it is a theoretical and conceptual paper and did not involve human participants, animal subjects, or the collection of primary data.

## Conflicts of Interest

The author declares no conflicts of interest.

## Data Availability

Data sharing not applicable to this article as no datasets were generated or analysed during the current study. This study is a conceptual and theoretical analysis. No primary data were generated or analysed.
